# The MIND model a microlearning AI-integrated instructional design for enhanced learning outcomes

**DOI:** 10.1038/s41598-025-24910-y

**Published:** 2025-11-20

**Authors:** Wali Khan Monib, Atika Qazi, Rosyzie Anna Apong, Jose Hernandez Santos, Malissa Maria Mahmud

**Affiliations:** 1 Centre for Lifelong Learning, Universiti Brunei Darussalam, Gadong, BE1410 Brunei Darussalam; 2 School of Digital Science, Universiti Brunei Darussalam, Gadong, BE1410 Brunei Darussalam; 3 Department of Chemistry, Universiti Brunei Darussalam, Gadong, BE1410 Brunei Darussalam; 4 School of Education, Sunway University, 47500 Selangor, Malaysia

**Keywords:** AI, Microlearning, Instruction design framework, Learning outcomes, Knowledge and skills improvement, Lifelong learning, Computer science, Information technology

## Abstract

In recent years, microlearning has gained significant attention due to technological advancements such as generative AI (GenAI), diverse learner needs, and a growing emphasis on improving learning outcomes. However, designing effective microlearning content remains challenging, as existing instructional design frameworks are inadequate for optimising learning outcomes. Therefore, this study developed a novel Microlearning AI-Integrated Instructional Design (MIND) Model to support effective instructional design to enhance learning outcomes. The model is grounded in multiple theoretical foundations, primarily the Technological Pedagogical Content Knowledge (TPACK) framework and the Factors Influencing Learning Outcomes (FIL) framework. To validate the MIND model, a mixed-methods approach was used with an intervention consisting of microlearning modules, comparing the MIND model (experimental group) with the ADDIE model (control group). Quantitative analysis using ANCOVA revealed that the MIND model significantly outperforms the ADDIE model in supporting effective instructional design to enhance learning outcomes, such as increased knowledge acquisition, understanding of module content, and improved knowledge application. Furthermore, t-test results indicated that learning outcomes within the MIND model group were consistent across gender, employment status, and locality, demonstrating inclusivity and accessibility. Kruskal–Wallis test showed a difference in learning outcomes across age groups, with younger learners achieving higher outcomes. Thematic analysis of qualitative data revealed that the modules developed using the MIND model featured media richness, interaction, engagement, self-concept, motivation, and satisfaction, contributing to learning outcomes. The MIND model serves as an innovative instructional design model that guides stakeholders in designing micro modules while remaining adaptable to conventional approaches, supporting flexible integration of cutting-edge technologies across formal, non-formal, and informal learning.

## Introduction

Microlearning has increasingly attracted the attention of researchers in recent years^1–3^. It is an instructional approach that delivers targeted, action-oriented, bite-sized content to achieve specific objectives within a short period^4^. Typically, this approach combines concise textual content with visuals, such as diagrams, images, or tables, and interactive micro-activities that promote learner engagement^5^. By presenting information in manageable segments^6^, microlearning facilitates more efficient and practical knowledge transformation^7^. This approach aligns with learners’ growing desire for autonomy, enabling them to decide what they learn, when they learn, and how they learn. The prevalence of short attention spans, together with the widespread use of smart devices and social media, further underscores the relevance of microlearning^8^.

Its rising adoption is largely attributed to the changing needs of learners, who increasingly demand flexible, accessible learning^9^. Furthermore, the increasing interest in microlearning is closely linked to the rise of social media, which, according to Taylor and Hung^1^, has transformed people’s information-consumption habits towards a single, discrete topic presented in a short duration. Platforms like X (Twitter), Instagram, TikTok, and YouTube Shorts have popularised the use of short-form content. For example, short tutorial videos have become a prevalent method of informal learning for numerous individuals^1^. Microlearning is applicable across various settings^3^, including workplace training and self-directed learning^10^ as well as formal education contexts^7^. Its concise nature allows content to be easily distributed across diverse media^11^.

Moreover, the increasing integration of AI in educational platforms has reinforced the significance of microlearning in delivering concise, learner-centred content. AI technologies can personalise learning experiences, automate feedback, and adapt content based on learner progress. However, research on how to systematically design and implement microlearning remains limited, and studies emphasise AI integration into microlearning instruction design^4^. Nevertheless, systematic guidance for designing microlearning while leveraging AI has received inadequate attention. Challenges persist in its design^12^ and form^13^, such as balancing conciseness with comprehensiveness^14,15^, avoiding content fragmentation^12^, and maintaining relevance across diverse topics. There is currently a lack of a widely accepted model integrating AI into microlearning instructional design, highlighting the need for a more structured approach. Therefore, this study aimed to develop the MIND Model to support effective instructional design and enhance learning outcomes.

## Literature review

### Microlearning

Prior studies have examined various aspects of microlearning, including user perceptions, instructional effectiveness, and design strategies. For example, Iqbal et al.^16^ explored postgraduate residents’ perceptions of microlearning environments, reporting overall satisfaction, with higher satisfaction among female residents, participants aged 25–30, and those in internal medicine. Similarly, Choo and Rahim^17^ demonstrated that microlearning can achieve outcomes comparable to face-to-face active learning in pharmacy education, while also offering flexibility, cost-effectiveness, and suitability for distance learning. This study examines key factors, including gender, age, locality, and employment status, in AI-enhanced microlearning, with the goal of determining whether they influence learning outcomes.

Other studies have focused on instructional design and efficacy. For example, Lee et al.^14^ evaluated a mobile microlearning course in journalism education, showing increased knowledge, decision-making confidence, and skill performance. The study highlighted areas for improvement, such as automated feedback, gamified exercises, and interactive content. Likewise, Dolowitz et al.^18^ found that mobile microlearning applications, such as NeNA, can enhance employee performance when iterative user feedback is incorporated. Moreover, Sankaranarayanan and Mithun^19^ examined AI-enabled microlearning in a database programming course, finding that students valued immediate feedback and simplified concepts but faced challenges with inconsistent or inaccurate responses for complex tasks. AI is essential, transforming how individuals learn, work, and interact^20,21^. Despite these advancements, designing effective microlearning experiences remains challenging^12^. Therefore, integrating AI in instructional design is critical for addressing the challenges and enhancing instructional effectiveness, particularly in diverse learner contexts.

### AI in instructional design

The integration of AI into instructional design is essential for effective instruction. It can be used to enhance the analysis of different learning needs, content design, and development. Traditional instructional designs often assume homogeneity, neglecting differences in prior knowledge, pace, or motivation. AI, such as GenAI, can be used to tailor microlearning content and feedback to individual learners, resulting in improved engagement and performance. It can help in generating content and selecting assessment strategies aligned with established taxonomies such as Bloom’s taxonomy^22,23^. This, while maintaining close human oversight, streamlines the design process and saves time. It supports resource scalability by producing diverse instructional materials in various media formats, including audio, video, and presentations. Evidence from multiple studies highlights these benefits. For instance, Kohnke et al.^24^ demonstrated that GenAI-focused microlearning modules enabled pre-service teachers to increase their confidence in lesson planning and the ability to adapt AI tools for differentiated instruction, formative assessment, and culturally responsive teaching. Similarly, Willenborg and Withorn^25^ demonstrated that a six-lesson microcourse on GenAI in college classrooms enabled students to efficiently apply AI in research and writing while accommodating instructors’ time constraints.

Moreover, Baillifard et al.^26^ illustrated that with a personal AI tutor for psychology students by modelling each student’s understanding of key neuroscience concepts and generating adaptive retrieval practice; students achieved up to 15 percentile points higher than peers without AI support. In a meta-analysis, Sun and Zhou^27^ found that GenAI interventions significantly improved college students’ academic achievement, with medium effect sizes, particularly when AI-supported tasks involved independent learning, text-based content, or smaller cohorts. Practical applications illustrate the importance of thoughtful instructional design when integrating AI. Ahlgren et al.^28^ demonstrated that AI effectiveness depends on structured guidance, tailored tasks, and contextualised application principles directly transferable to instructional design in education.

GenAI can enhance microlearning design by enabling the creation of concise content in the form of videos, podcasts, and images—allowing learners to engage more deeply with material; however, challenges remain, including platform compatibility issues, fragmented learning for complex topics, and reduced social interaction, which necessitate careful educator strategies and human facilitation to maintain collaborative and social aspects of learning^29^. There is limited work on developing an instructional design framework that provides educators with detailed guidance for the practical development of microlearning instruction, particularly when integrating AI to enhance learning outcomes. Therefore, this study supports interactive microlearning instructional design responsive to diverse learners’ needs.

### Microlearning instructional design

As education continues to evolve, novel instructional design models are essential for effective instructional design. Traditional instructional design models are increasingly proving ineffective and less adaptable in this rapidly changing world^30^. Factors such as shortened attention spans, widespread smartphone usage, and easy access to information through search engines have contributed to the diminishing relevance of these models^31^.

There are models, such as the ADDIE (Analysis, Design, Development, Implementation, and Evaluation) model, and the microlearning model. However, while these models are valuable, they are subject to several limitations. The ADDIE model, a colloquial term for a systematic approach to instructional development, lacks academic rigour and a single author, having evolved informally through oral tradition^32^. It is further stated that anyone is free to attribute their own interpretations and characteristics to the model as they may want. Traditional models, such as ADDIE, are challenging to apply in dynamic and adaptive educational contexts and are increasingly considered inapplicable in the modern technology-based era^33,34^. In addition, it is costly and requires adequate funding for implementation^33,35^. Moreover, it is also criticised for being linear, hierarchical, and time-intensive^36^. It faces challenges in adapting to rapidly changing educational contexts and integrating emerging technologies. Another model is proposed by Dolasinski and Reynolds^31^, a learning model that integrates microlearning in four phases: (1) predevelopment of the learning, (2) development and delivery of learning content, (3) learner participation and practice, and demonstration of activity, and (4) evaluation. However, this model also has significant limitations. First, it lacks empirical validation; that is, the model has not been tested for its effectiveness in real-world settings. Without empirical validation, its practical applicability and impact may be uncertain. Second, its generalisability is limited, as the inquiry is based on past research that is limited to workplace learning and theories^31^. As a result, its applicability to broader educational domains may be limited. Challenges in designing microlearning experience persist, as highlighted by recent studies^37–39^. Therefore, to overcome the existing challenges, this study developed the MIND model to support effective instructional design.

### MIND model development

The novel MIND Model is developed to support effective instructional design through the flexible integration of cutting-edge technologies, including but not limited AI, to enhance learning outcomes. The novelty of the Model can be identified across several dimensions. First, it focuses on educators and learners’ needs, explicitly emphasised during the analysis stage. Other needs can be considered depending on the context, such as institutional requirements, technological infrastructure, curriculum standards. Second, the model is based on robust theoretical foundations, particularly the TPACK and the FIL frameworks. TPACK, introduced by Mishra and Koehler^40^, emphasises the interconnectedness of content knowledge (CK), pedagogical knowledge (PK), and technological knowledge (TK) in teaching. This is particularly important in microlearning, where content must be concise, requiring careful design considerations^41^. For example, educators need to understand influential factors, including PK and CK, and know what content to modularise, how much to modularise, and how to adjust delivery strategies. The study incorporates Situational Awareness (SA) into the TPACK framework for educators’ side, resulting in a conceptual extension of TPACK as SATPACK. This extension emphasises the importance of being aware of ongoing dynamics within the learning environment. Furthermore, it integrates the FIL framework to guide content design, development, and delivery. FIL framework is developed by Monib et al.^41^ and emphasise the importance of contextual (media richness), behavioural (interaction and engagement), cognitive (comprehension), and affective (motivation, self-concept, and satisfaction), influencing learning outcomes drawing from multiple theories such as the Expectancy-Disconfirmation, constructivism, Self-determination, Situational Awareness, Cognitive Multimedia Learning, and Andragogy. Together, these frameworks shape a comprehensive, theory-informed foundation for the MIND Model. Third, it is learner-centred, prioritising learners’ needs, enabling them to take responsibility for self-paced learning. Fourth, it uniquely separates the delivery stage (where the educator presents content) and the receiving stage (where learners receive, practice, interact, and engage with content, peers, and educators), emphasizing active learning, unlike ADDIE, which treats instruction as a single, passive implementation stage. This distinction is essential in the current MIND Model, which highly emphasises factors such as interactivity and engagement. The ADDIE model pays limited attention to interaction, such as learner-educator, learner-content, and learner-learner, in content delivery and has been criticised by constructivists. Bates^42^ states

ADDIE model is what might be called ‘front-end loaded’ in that it focuses heavily on content design and development, but does not pay as much attention to the interaction between instructors and students during course delivery. It has been criticised by constructivists for not paying enough attention to learner-instructor interaction, and for privileging more behaviourist approaches to teaching.

In contrast, MIND model emphasises meaningful interaction with content, educators, and peers, considering TPACK^40^ and FIL frameworks^41^. This interaction is critical, as delivery without reception, such as learners merely receiving content without interaction, may not produce meaningful learning outcomes. Fifth, the MIND Model is outcome-oriented, with all stages and sub-elements systematically aligned to achieve specific learning outcomes. The MIND model integrates both assessment, which measures learning outcomes, and evaluation, which evaluates course’s overall effectiveness and quality, including teaching, content, delivery, and learning experience. In the ADDIE model, evaluation tests the curriculum rather than individual learning, reflecting its curriculum-oriented nature. Evaluation does not explicitly measure learning outcomes and operates at the macro level, judging the quality or effectiveness of a programme, course, or curriculum (e.g., a committee reviewing a new curriculum after its first year of implementation, an educator receives feedback on a course at the end of the semester). As Peeters and Schmude^43^ clarify the difference,

Learning assessments from students are different from programmatic or curricular evaluation. While education disciplines…use assessment to mean assessments of students’ learning, it seems that many in American pharmacy education…use assessment to refer to program evaluation. This misuse of the term assessment for program evaluation seems unfortunate. There are textbooks and internet-based resources on program evaluation.

Their observation underscores the importance of maintaining conceptual clarity between assessment & evaluation to avoid conflating learner-level outcomes with programme-level judgments. This distinction is reinforced in institutional practices. For example, Colorado College differentiates the two in its guidelines, defining assessment as focused on student learning outcomes and evaluation as addressing broader departmental, programmatic, or administrative concerns^44^. The distinction is also recognised in scholarly publishing. The Springer journal *Educational Assessment, Evaluation and Accountability*^45^ differentiates the two, both in its title and its aims and scope, stating:

The main objective of this international journal is to advance knowledge and dissemination of research on and about assessment, evaluation and accountability of all kinds and on various levels as well as in all fields of education.

**Fig. 1:**
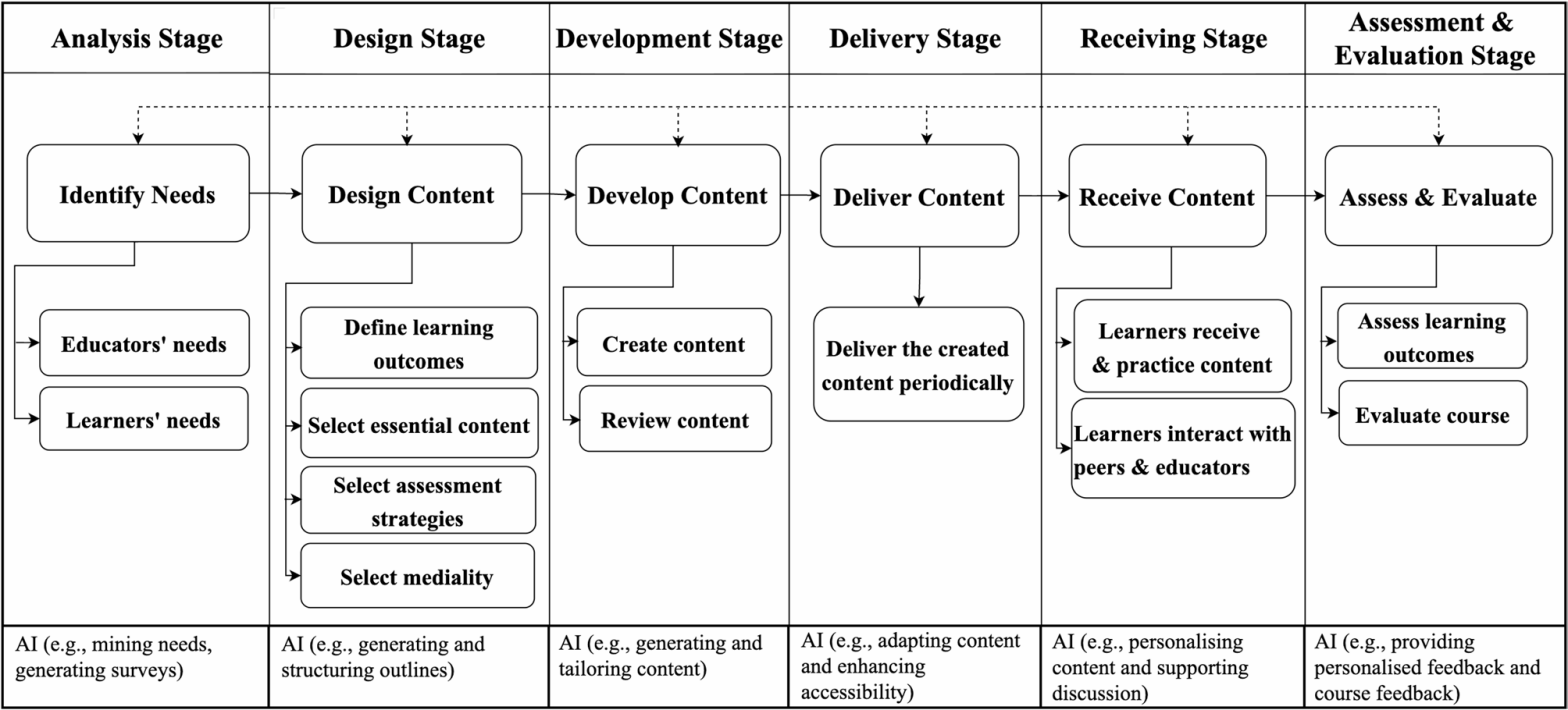
The MIND model.

Sixth, it is cost-effective and time-efficient; unlike other models, which require extensive resources and multiple stakeholder involvement. Seventh, the model is grounded in microlearning principles, with AI integration supporting all stages—from needs analysis to content creation, content delivery, and content receiving, and assessment & evaluation—reducing resource demands while ensuring quality instructional design. Eighth, the MIND model is non-linear, where each stage is interconnected. If there is a need for improvement, educators can address and return to earlier stages. Lastly, it can be adopted across formal, non-formal, and informal learning settings. The model consists of six stages: Analysis stage, design stage, delivery stage, receiving stage, and assessment & evaluation stage. Certain stages of the MIND Model can be adapted to suit different learning settings—formal, non-formal, or informal—where, for example, assessment may remain essential in formal contexts but could be optional or modified in non-formal and informal learning (see Fig. 1).

#### Analysis stage

The analysis stage involves examining the needs of both educators and learners. For educators, this includes assessing their preparedness to design microlearning experiences and identifying any required professional development. AI can support the identification of educators’ and learners’ needs, while human oversight and interpretation remain crucial. AI supports educators in assessing learners’ needs through multiple strategies, including learner profiling, needs mining, surveys, and diagnostic assessments to uncover knowledge gaps, skills, preferences, and challenges. For example, AI can analyse large volumes of data from students’ interactions, assessments, and learning preferences to determine existing knowledge and knowledge gaps^46^. Similarly, machine learning algorithms can cluster learners by proficiency or learning style and predict skills or concepts they may struggle with. Moreover, GenAI can assist in creating surveys to identify the needs of educators and/or learners. All identified needs should remain within the course scope and align with institutional policies. The principle of andragogy, which emphasises learners’ ‘need to know,’ should guide educators not only during the need analysis stage—ensuring that they address the why, what, and how questions^47^. Additionally, individual, situational, and subject differences, as well as other factors, should be considered. Overall, learners’ needs can then be translated into specific learning outcomes in the subsequent stage.

#### Design stage

The design stage involves designing microlearning content for formal, non-formal, or informal learning, following the analysis stage. Clear, specific, and measurable learning outcomes should be defined in alignment with Bloom’s Taxonomy^22,23^ or any other taxonomy, ensuring that both cognitive depth and skill application are systematically assessed. These outcomes guide the selection of essential content, ensuring both relevance and clarity. The appropriate assessment strategies should be planned in alignment with the learning outcomes and may include formative and summative assessments. GenAI can aid in the design stage by outlining the module including learning outcomes, selecting instructional content, and assessment & evaluation strategies, and other related elements, while ensuring alignment across all components. These elements can be revisited in subsequent stages as needed for refinement. Aligned with this, the mediality can be selected, including the delivery mode (e.g., online, face-to-face, or blended)^48,49^, as well as the media formats, such as videos, podcasts, presentation slides, and infographics. In mediality, selecting an appropriate microlearning platform is particularly crucial, as it facilitates effective content design and enables learners to navigate and engage with the modules meaningfully in later stages. In addition, it is crucial to consider other factors—such as time constraints, institutional policies, and factors identified at the analysis stage. Overall, a well-designed module ensures alignment across all components.

#### Development stage

The development stage involves creating and reviewing microlearning content, following the design stage. Content can be created in different media formats, such as short videos, handouts, or presentation slides, ensuring alignment with the intended learning outcomes and assessment strategies. Educators—having a clear understanding of the content and learning outcomes—can efficiently develop appropriate assessment & evaluation strategies, which can be revisited and refined in later stages if needed. Educators should consider various factors, including but not limited to content-specific factors such as media richness, interactivity, engagement, comprehension, self-concept, motivation, and satisfaction^41^. Instructional content should include interactive elements, e.g., quizzes and simulations, to foster active learning and provide opportunities for practical application^6,50,51^. To support diverse learners’ needs, content should be multi-sensory and multi-modal^31,52^, incorporating rich media such as visuals, graphs, charts, audio, and video to enhance engagement and comprehension. Modules should be presented in bite-sized segments to prevent cognitive overload and support sustained engagement^3^. The time is relatively short, subjective^49^, and can be a few seconds to a few minutes in duration, offering just-in-time access^4,53^. When creating bite-sized content, such as a video, ensure the content is scripted, produced, edited, and reviewed to maximise clarity and learning impact. Zhang and West^75^ state that content refinement often requires iterative revision to eliminate non-essential information. Therefore, creating a micro module remains particularly challenging in microlearning instructional design. In this context, GenAI can be used to mitigate this challenge by generating content, refining content, and efficiently condensing materials into digestible microlearning units^3,54^ as well as assessment strategies that align with learning outcomes. It further enables tailoring of content to individual learners’ needs, while considering the FIL framework. At this stage, if modules are designed for formats other than microlearning, the content size can be adjusted accordingly to suit the intended learning context. Overall, the development stage ensures that instructional materials are carefully designed, resulting in modules to support effective learning experiences.

#### Delivery stage

The delivery stage involves delivering the created content periodically, following the development stage. Microlearning content can be delivered through face-to-face^48,49^, online^12,48^, and/or blended mode^12,48,55^, aligned with the mediality specified during the earlier stage. In practice, microlearning is most often delivered online, given its flexibility and scalability. Regardless of the delivery of microlearning, AI-enabled platforms and formats play a crucial role in ensuring flexibility and accessibility for diverse learners. At this stage, AI can enhance microlearning by personalising content to individual needs, preferences, and pace, and by recommending adaptive learning paths based on performance. It can also automate notifications and reminders to keep learners on track, while providing accessibility features such as text-to-speech, translation, and alternative formats. Other learning management systems (LMS), educational apps, digital tools, and printed materials can also be used to deliver microlearning^12,14,55–57^. Based on learners’ needs, content can be delivered through diverse media such as bite-sized videos^12,18^, infographics, podcasts^12,18^, and so on. The timing and frequency of delivery remain flexible, ensuring that learning materials are accessible in a timely and aligned with learners’ pace. Overall, this stage ensures content is delivered in a timely while maintaining flexibility to accommodate individual schedules and learning paces.

#### Receiving stage

In the receiving stage, learners receive and practice the content. This stage emphasises active learning, engaging learners as they interact with the educator, peers, and content to develop understanding and skills. Learners engage with multiple forms of content—such as learning materials, scheduled quizzes, and interactive activities that encourage practice and reinforce learning. For example, while interacting with microlearning content, learners receive concise instructions from the educators, followed by a practical task such as using an AI tool to summarise a passage, enabling them to practice and reinforce their understanding. Learners interact with AI-driven exercises, quizzes, and simulations, receiving personalised, adaptive feedback and guidance on applying concepts, fostering autonomous, self-directed learning. AI analyses learners’ behaviour patterns, predicts potential academic issues, and facilitates timely interventions to enhance performance^58^. A key feature of this stage is learner autonomy, where learners select from the available modules and engage with them at any time, from any location, and via any device, such as a laptop, mobile phone, promoting flexible, accessible, and inclusive learning^3^. Overall, this stage ensures that learners can engage with and practice the content in a meaningful and flexible manner.

#### Assessment & evaluation stage

The assessment & evaluation stage involves assessing learning outcomes and the effectiveness of the course, following the receiving stage. Educators assess learning outcomes through the assessment strategies selected at the earlier stage, such as formative and summative assessments. These assessments serve the purpose of assessing learners’ knowledge, skills, and competencies. Formative assessment aims to provide ongoing feedback^59^, enabling learners to monitor their progress, identify areas for improvement, and refine their work for better learning outcomes^60^. In contrast, summative assessment, being graded, assesses overall achievement at the end of a learning period^61^. In this way, formative assessment supports growth and preparation, while summative assessment validates attainment. GenAI can assist educators in recommending personalised feedback supporting improvement during formative assessment. AI can simultaneously enhance automated grading, ensuring greater efficiency, consistency, and objectivity. For instance, a study by Gao et al.^62^ found that DeepSeek, an AI tool, achieved higher reliability, provided more relevant feedback on content, language use, organisation, and coherence, and was useful for enhancing English as a Foreign Language (EFL) writing assessment. Assessment measures learners’ learning outcomes, while evaluation considers evaluators’ perspectives on a course overall effectiveness and quality. For example, learners reflect on the effectiveness of teaching and the quality of the content during the course or at the end. Educators use this feedback to identify areas for improvement. This iterative process enhances instructional quality, promotes effective learning, and increases learner satisfaction. Overall, the MIND model stages are interconnected, allowing continuous refinement, flexibility, and non-linear progression throughout the instructional process.

## Methodology

### Research design and materials

This study employed a mixed-methods approach to validate the MIND Model through the intervention, in which the educator developed microlearning modules, delivered them to learners, and assessed subsequent learning outcomes. The research was conducted at C3L, UBD, with ethical approval granted by the C3L Ethics Committee on 21 December 2023, and all procedures followed relevant guidelines and regulations. Participants, comprising educators and learners, provided informed consent, confirming their voluntary participation and acknowledging their right to withdraw at any point without consequence. Learners were randomly selected, and only those who consented to participate in both the pretest and posttest were included in the study. They were granted free access to the course, which comprised a series of micro-modules. Confidentiality was maintained throughout the study.

The intervention used microlearning modules, with the experimental group applying the MIND model and the control group the ADDIE model (Table 1). For the experimental condition, educators first participated in workshops to address their needs identified during the MIND Model’s needs analysis stage, enhancing their skills in designing, developing, and delivering microlearning modules. Subsequently, one educator voluntarily designed and developed a course, Mastering AI-Powered Research Skills, consisting of 12 micro-modules, following the MIND Model design and development stages. The course was selected to reflect the evolving learning paradigm^63,64^ and to address learners’ needs identified at the analysis stage.

**Table 1:** Mastering AI-powered research skills.

No	Microlearning Modules
1.	Overview: What is AI, and Importance for Research
2.	Core AI Concepts for Research
3.	AI-assisted Research Skills
4.	Integrating AI into Research
5.	Search Optimisation with ChatGPT and Google Scholar
6.	Leveraging ChatPDF and Wordtune for Enhanced Reading
7.	Unlocking Research Potential with Perplexity
8.	ChatGPT Channels for Data Analysis Insights
9.	Creating Visual Figures with Napkin
10.	Understanding Ethical Principles in AI Research
11.	Bias and Fairness in AI
12.	Privacy and Data Protection

A range of AI-powered tools was employed by the educator to develop the microlearning modules, guided by the FIL framework, including media richness, interaction, engagement, comprehension, motivation, self-concept, and satisfaction^41^. The tools included ChatGPT, Napkin AI, Gamma, NoteBookLM, and CapCut, and may be used alongside other emerging GenAI tools for designing microlearning modules in the future. The modules were delivered via the AI-enabled OpenLearning platform in multiple formats—videos, podcasts, presentation slides, and PDFs—to accommodate diverse learning needs. A registration link was made available to learners, enabling them to sign up and access the modules.

In the receiving stage, learners received a link to the platform, through which they signed up and enrolled in the microlearning modules. Afterward, they accessed the modules, actively engaged with the content, and practiced the activities, while platform analytics tracked their progress and facilitated interactions with the educator. Following the MIND Model’s assessment & evaluation stage, pre- and post-tests were administered, and learners’ reflections were collected to evaluate the effectiveness of the intervention in improving learning outcomes.

In the control condition, the educator developed the same course following the ADDIE model without using AI tools. During the analysis stage, learners’ needs were identified. In the design and development stages, instructional materials—including videos, handouts, and supplementary resources—were created to address the same content areas as the experimental condition. The implementation stage consisted of blended delivery, combining face-to-face sessions and access through Canvas LMS. Consistent with ADDIE, the evaluation stage primarily focused on course quality. However, to allow a direct comparison with the experimental condition, pre- and post-tests were administered in the control condition. This enabled assessment of learners’ improvement in learning outcomes.

The pre-test was administered to both the experimental and control groups to assess baseline knowledge prior to accessing the microlearning modules. After five weeks, the post-test was distributed to the same learners to measure learning outcomes. The pre- and post-tests consisted of 12 multiple-choice questions. These questions were categorised into three domains: knowledge of GenAI (Q1–Q3), applications of AI in research (Q4–Q8), and ethical considerations related to AI (Q9–Q12).

For qualitative insights, participants were asked to provide written reflections on their experience with the microlearning modules. They were specifically prompted to reflect on key content features such as media richness, interactivity, engagement, satisfaction, and comprehension, as well as to evaluate the impact of the modules on their knowledge and skills development. The reflection prompt read: *‘Write your reflection about your experience of microlearning modules. Reflect on content factors such as media richness, interactivity, engagement, satisfaction, and comprehension. Consider whether it helped improve your knowledge and skills.’* To ensure content validity, two academic experts from Universiti Brunei Darussalam independently reviewed and validated the microlearning modules, the pre- and post-tests, and the reflection prompts.

A total of 64 learners completed both the pre-test and post-test, with 32 in the experimental group and 32 in the control group. In the experimental group, 17 females and 15 males. Age distribution showed that 15 participants were aged 35–44, 10 were 25–34, and 7 were 18–24 years old. Twenty-five participants were employed, while 7 participants were non-working. In terms of residence, 28 participants lived in urban areas, and 4 participants resided in rural areas. In the control group, 13 females and 19 males. Age distribution comprised 10 participants aged 18–24 years, 14 aged 25–34 years, and 8 aged 35–44 years. Ten participants were working, and 22 were not. Regarding residence, 30 participants from urban areas and 2 from rural areas.

### Analysis

This study analysed learners’ quantitative data from pre- and post-tests alongside qualitative data from their reflections to validate the MIND model (experimental group) and enable comparison with the ADDIE model (control group). Quantitative data were analysed using SPSS. An ANCOVA was conducted to compare post-test scores between the experimental and control conditions while controlling for pre-test scores. Prior to analysis, assumptions for ANCOVA and t-tests were satisfied. For ANOVA, the normality assumption was not met; therefore, a Kruskal–Wallis test was conducted. For the experimental group, further analysis included independent-samples t-tests to examine differences across participants’ gender, employment status, and locality, where the normality assumption was met. Differences across age groups were assessed using a Kruskal–Wallis test.

For qualitative data analysis, ATLAS.ti was used to facilitate thematic coding. Participant reflections were first cleaned and reviewed multiple times to ensure data familiarity, then imported into ATLAS.ti for systematic coding following a structured process^65,66^. Initial codes were generated to capture key features relevant to the research questions, considering both explicit and implicit meanings. Codes were examined for patterns, and similar ones were grouped into potential themes, which were then refined for internal consistency and distinction, and cross-checked against the data to ensure accuracy. Each theme was clearly defined and named to maintain conceptual clarity and alignment with the research focus. The analysis was finally written up using illustrative quotes.

Several measures were implemented to enhance the reliability and validity of the qualitative analysis. To establish intercoder reliability, a second coder independently analysed a portion of the dataset using the coding framework developed by the primary researcher. For inter-rater reliability, the codes were compared, and the level of agreement was measured using Cohen’s kappa, yielding a value of *κ* = 0.689, *p* = .01, indicating substantial agreement^67^, supporting the reliability of the thematic analysis^68^.

Discrepancies were resolved through discussion and consensus, with refinements made to code definitions as necessary, ensuring rigour across the full dataset. To enhance credibility, thick descriptions and direct participant quotes were incorporated to provide transparency and contextual depth. The quantitative findings were complemented and explained by qualitative evidence and further validated through comparison with existing literature.

## Results

This section presents findings, including quantitative results from inferential statistical analysis and qualitative insights from thematic analysis.

### Inferential statistics

Learners’ performance was assessed before and after the intervention to evaluate the effectiveness of the MIND model and compare it with the ADDIE model. The ANCOVA was conducted to compare post-test scores between the MIND and ADDIE groups, controlling pre-test scores. Descriptive statistics indicated that participants in the experimental condition who learned through the MIND model (M = 86.000, SD = 6.932) scored higher on the post-test than those in the control condition who learned through the ADDIE model (M = 79.312, SD = 5.038). Table 2 indicates the pre-test covariate was a significant predictor of post-test scores, F(1, 61) = 104.519, *p* < .001, partial η^2^ = .631. After adjusting for pre-test scores, the group effect remained significant, F(1, 61) = 45.976, *p* < .001, partial η^2^ = .430. This indicates that participants in the MIND model group outperformed those in the ADDIE model group, supporting effective instructional design to enhance learning outcomes. The overall model explained a substantial proportion of variance in post-test scores, R^2^ = .720, adjusted R^2^ = .710.

**Table 2:** ANCOVA comparing MIND and ADDIE instructional design effectiveness.

Source	SS	df	MS	F	*p*	Partial η^2^
Pretest (covariate)	1437.762	1	1437.762	104.519	***	.631
Intervention (MIND vs ADDIE)	632.445	1	632.445	45.976	***	.430
Error	839.113	61	13.756			

***p < .001.

For the experimental condition, to examine differences in learning outcomes based on gender, age, employment status, and locality, independent samples *t*-tests and a Kruskal–Wallis Test were conducted. First, change scores were calculated for each learner by subtracting their pre-test score from their post-test score. The mean change scores were then compared across demographic groups. An independent samples *t*-test revealed no significant difference in learning outcomes between males (M = 12.400) and females (M = 13.117), *t*(30) = − 0.497, *p* > .05 (see Table 3). Similarly, no significant differences were found between urban (M = 12.607) and rural (M = 14.000) participants, *t*(30) = − 0.640, *p* > .05, or between working (M = 12.240) and non-working participants (M = 14.714), *t*(30) = -1.462, *p* > .05.

**Table 3:** T-test across gender, locality, and employment status.

	Group	N	M	t	p	df
Learning outcomes	Male	15	12.400	− .497	ns	30
	Female	17	13.117			
	Urban	28	12.607	− .640	ns	
	Rural	4	14.000			
	Working	25	12.240	-1.462	ns	
	Not working	7	14.714			

ns = not significant.

To compare differences among age groups, a Kruskal–Wallis test was used since the assumption of normality was not met. The results indicate a significant difference in learning outcomes across age groups, χ^2^(2) = 8.495, *p* < .05 (see Table 4). Following this significant result, Mann–Whitney U test was conducted where the results indicated that participants aged 18–24 scored significantly higher than those aged 35–44 and 25–34 (18–24 vs. 35–44; 25–34, *p* < .05), while differences between other groups were not significant (25–34 vs. 35–44, *p* > .05). This difference may be attributed to younger learners being more technologically savvy, enabling them to engage more effectively with microlearning environments.

**Table 4:** Kruskal–Wallis and post hoc Mann–Whitney U tests across age groups.

	N	Kruskal–Wallis Test				Mann–Whitney U		
		Age	Mean Rank	χ^2^ (df)	*p*	Comparison	U	*p*
Learning outcomes	7	18–24	24.360	8.495(2)	*	18–24 versus 35–44	18.000	*
	10	25–34	17.600			18–24 versus 25–34	14.500	*
	15	35–44	12.100			25–34 versus 35–44	43.500	ns

*p < .05; ns = not significant.

### Thematic analysis

Thematic analysis of reflections from the experimental group corroborated the quantitative findings, showing improvements in key learning outcomes—knowledge acquisition, comprehension, research skills, and problem-solving—while also identifying influential factors (see Table 5). Learners reported that microlearning was effective in enhancing their understanding and use of AI tools in academic contexts. Many participants expressed increased confidence in applying the knowledge and skills gained to real-life situations. For example, one participant reflected:

The module content was quite clear and easy to comprehend in a short time. I have gained knowledge that made me feel confident about GenAI and scientific resources. I can say that now I feel that I can easily refine the query, develop a clear understanding, and then apply it practically. [It] will surely help me while making my final year project reports, etc. Although I need further practice to master the skills, I feel that I learned more from it.

**Table 5:** Themes and codes.

Theme	Codes	
Learning outcomes	Improved knowledge acquisition	Improved problem-solving skills
	Enhanced comprehension	Knowledge application
	Improved research skills	
Media richness	Well-organised content	Concise text
	Coherent media	Enough visuals
	Informative graphs and tables	clear visuals
Interaction	Interaction with educators	Exercises
	Asking questions	Quizzes
	Visual aids	Games
Engagement	Various learning styles	Simple
	Variety of media	Relevance
	Short	Reflections
	Interesting	
Comprehension	Easy to understand	Well-organised
	Effective presentation	Well-structured
	Clear examples	Concise information
Self-concept of the learner	Self-directing learning	Autonomy
	Plan own learning	Self-reflection
Motivation to learn	Goal achievement	Personal growth
	Rewarding	Curiosity
	Task completion	Accessible
Satisfaction	Less cognitive overload	Interesting
	Easy to access	Informative
	Easy to follow	Compact
	Easy to recap	Media-rich content
	Learn own pace	Unique
	Easy to understand	Enjoyable

Another participant reflected positively on the overall learning experience, stating, *‘Overall, the module was comprehensive and enhanced my knowledge and skills in AI-assisted research.’* Across reflections, learners reported enriched experiences in several areas, including media richness, interactivity, engagement, comprehension, satisfaction, motivation to learn, self-concept, and accessibility. Learners responded positively to the media richness, highlighting the use of clear visuals, informative graphs and tables, well-organised content, and concise text.

In terms of interactivity, participant feedback generally aligned with the quantitative findings—learners considered the content to be interactive. Interactive elements included interaction with the educator, verbal components (Q&A), non-verbal elements (games), and activity-based tasks (such as short exercises and quizzes). Visual aids further enhanced interactivity, while accessible communication channels contributed to a supportive learning environment. However, some learners noted limited peer interaction, particularly during online sessions. As one learner shared, *‘In the online class, I couldn’t interact with my peer.*’

Moreover, many participants found the modules engaging, citing the relevance and interest of the content, the use of a variety of media, the support for effective learning, the accommodation of diverse learning styles, and simple and short content. One learner remarked, *‘I felt engaged due to the variety of media usage, and I found the short content relevant.’*

Regarding comprehension, learners reported that the modules were easy to understand, attributing this to the inclusion of clear examples, effective presentation, a well-organised, structured format, and concise information. Furthermore, the self-concept of the learner involves exercising autonomy and self-directed learning. Several participants noted that microlearning encouraged independent learning and peer socialisation, as illustrated by the comment: *‘Microlearning is short and is standalone to learn independently.’*

Learners’ motivation to learn was driven by a sense of goal achievement, personal growth, curiosity, accessible learning materials, and successful task completion. Learners were satisfied, reporting that the microlearning module was easy to follow, access, recap, and understand. They were satisfied because they found the approach unique, enjoyable, and interesting. They were also satisfied experiencing less cognitive overload, appreciating its short, informative, and media-rich content. For instance, one participant stated, *‘I appreciate the short video and am happy to have gained useful insights.’* Similarly, another participant reflected, *‘I like the short and informative nature of the video, and I feel it [was] effective. I am quite satisfied with the provided practical examples, and the module met my content and delivery format expectations.’*

In addition, they acknowledged the feasibility of the microlearning module, highlighting its flexibility and accessibility anytime and anywhere through any device. One participant mentioned, *‘[In] terms of convenience, I can easily access it through my mobile or laptop and watch it multiple times, and, side by side, I can practice it too.’* Suggestions included more collaboration opportunities and interactive activities simulating real scenarios.

## Discussion

The findings of this study revealed the effectiveness of the MIND Model in instruction design to enhance learning outcomes. The educator followed all stages of the model, from needs analysis to the design and delivery of microlearning modules, through the receiving, and assessment & evaluation. The ANCOVA results indicated that the instructional design developed using the MIND model led to significantly higher post-test scores than the instructional design developed using the ADDIE model. This suggests that the MIND model has significant effectiveness in improving learning outcomes, including knowledge, comprehension, and application. Based on Bloom’s taxonomy, this indicates improvements not only in lower-order outcomes but also in higher-order outcomes.

The overall model explained 72% of the variance in post-test scores, demonstrating a large and practically meaningful effect. Similarly, studies comparing ADDIE with other models, such as SAM, have reported lower performance for ADDIE. For example, the study by Ali et al.^69^ highlights the limitations of ADDIE-based instruction. Their comparison of SAM and ADDIE in STEM teaching showed higher post-test gains and better conceptual understanding for SAM, reflecting ADDIE’s lower engagement and limited interactivity.

Consistent with prior research on microlearning, which has reported increased knowledge, retention, higher-order thinking, professional competencies, and/or learning performance^70–74^, this study extends the literature by demonstrating the effectiveness of AI-enhanced microlearning designed according to the MIND Model. In a systematic literature review on microlearning, Monib et al.^3^ identified a range of learning outcomes categorised according to Bloom’s Taxonomy, such as knowledge acquisition, retention, recall, improvement, transfer, and application; higher-order skills such as critical thinking, problem-solving, feedback literacy, and self-regulation; professional competencies including digital and pedagogical competence; and performance outcomes such as test results. This study advances the literature by focusing on AI-enhanced microlearning and leveraging GenAI tools within instruction design based on the MIND model to improve learning outcomes, consistent with prior research highlighting the effectiveness of AI tools in enhancing learning gains^70^.

Furthermore, demographic analysis revealed no significant differences in learning outcomes by gender, employment status, or locality, suggesting the broad inclusivity and accessibility of the instruction design developed based on the MIND model. All age groups demonstrated improvement, with younger learners showing particularly significant gains, which may reflect their digital fluency and familiarity with AI-based platforms. Therefore, although microlearning can be broadly inclusive, targeted digital scaffolding is necessary to support less digitally savvy learners while considering their age**.** Despite the overall success, learners identified areas for improvement**,** particularly related to peer interaction and video pacing. As Zhang and West^75^ note, designing microlearning environments that balance efficiency with meaningful engagement remains a challenge.

The integration of AI tools such as ChatGPT, Gamma, Napkin AI, and NoteBookLM reflects TK, while the learner-centred, scaffolded presentation of the modules demonstrates PK. This suggests that the educator was aware of emerging technologies, following the training and has chosen a GenAI topic that learners need more than ever. The careful design, development, and delivery of the module demonstrate the educator’s CK, TK, and PK. Considering SA alongside TPACK is therefore instrumental in meeting learners’ expectations. This confirms that effective technology integration must go beyond its adoption and consider the situational context.

Microlearning was found to be flexible and accessible. The feasibility of microlearning is also reported by Gross et al.^76^, who conducted a study on delivering Crew Resource Management training in 15-min segments. This may be one of the reasons for its growing adoption in various contexts, particularly mobile-based microlearning, which is increasingly integrated into various instructional contexts^77^.

Similarly, the findings from qualitative reflections supported quantitative findings, where learners reported improvements in areas such as knowledge acquisition, comprehension, and application, research, and problem-solving skills. The microlearning modules were considered contextual (media richness), cognitive (comprehension), behavioural (interaction and engagement), and affective (motivation, self-concept, and satisfaction) factors. This indicates that effective instruction designs should consider these crucial factors. The authors concluded that, when guided by a robust instructional design, including the MIND Model, microlearning offers scalable and inclusive education, reaching learners across various demographics, including gender, employment status, and location, at minimal cost. The findings support SDG 4 by promoting equitable and quality education, SDG 5 by ensuring gender-inclusive learning opportunities, and contribute to Wawasan Brunei 2035 goal 1, which aims to have the people of Brunei Darussalam educated, highly skilled, and accomplished. The MIND Model is particularly important as it provides a detailed description of each stage to design microlearning that maximises learning outcomes while addressing diverse learners’ needs. Furthermore, the MIND model integrates AI and microlearning, but is flexible enough to accommodate conventional learning; regardless of the format, the stages—from analysis to outcomes—remain consistent.

The ADDIE model is predominantly curriculum-oriented and does not explicitly focus on monitoring learners’ progress, limiting educators’ ability to optimise learning outcomes. Shakeel et al.^35^ argue that the ADDIE model requires adequate funding and a longer implementation timeframe. It cannot meet the demands of modern technology-based education^36,78^ and faces challenges in rapidly changing digital learning environments and in integrating cutting-edge technology, such as AI. Using ADDIE for microlearning is particularly challenging, as microlearning instructional content needs to be concise, immediately applicable, and responsive to diverse learners’ needs. Furthermore, involving multiple stakeholders can prolong the development process, increasing the risk that the content becomes outdated before it is used and undermining instructional relevance. In contrast, the MIND model leverages AI to be cost- and time-efficient, responsive to immediate learner needs, and both learner-centred and outcomes-oriented.

Following the ADDIE model in the instructional design was time-consuming. Its rigid and linear structure limited flexibility in developing and delivering materials to cater to diverse learner needs and hindered the integration of additional resources, such as AI tools. ADDIE, along with traditional models, has limitations such as restricted customisation, long design cycles, and limited flexibility^34^. This shows that the ADDIE model is problematic for both microlearning and traditional contexts due to several limitations discussed. In contrast, the MIND model required less time for instructional design, enabled faster implementation at lower cost, and allowed greater flexibility across stages, supporting effective instructional design and improved learning outcomes.

Overall, the findings provide compelling evidence for the effectiveness of the MIND model relative to the ADDIE model. Systematically implemented, the MIND model supports learner-centred, outcomes-oriented instructional design that aligns curriculum with intended learning outcomes. Compared with the ADDIE model, the MIND model outperformed, highlighting the model’s practical value for effective and responsive instructional design.

## Implications, limitations, and future research

The study has several crucial implications. Theoretically, the MIND Model extends instructional design theory by embedding situational awareness within TPACK (SATPACK), contributing to a learner-centred and outcome-oriented approach. This provides an in-depth understanding of how contextual factors, such as learners’ needs, learning environment, individual and situational differences, influence learning outcomes. In addition, the model is learner-centred and outcome-oriented, prioritising diverse learners’ needs and ensuring that all instructional decisions—from content design to assessment—are aligned with achieving specific outcomes. It distinguishes the implementation stage into delivery and receiving stages, unlike ADDIE, which treats implementation as a single passive process. Practically, the model provides educators with structured guidance to design instructional content. For institutions, it offers a cost-effective approach that enables the implementation of microlearning without excessive resource investment and is applicable across blended, online, and face-to-face modes in formal, non-formal, and informal learning settings.

Attention should be paid to design quality, particularly in ensuring content clarity and appropriate pacing. It is crucial for educators to focus on human-centred AI integration that helps rather than overwhelms learners. Designers should also explore integrating collaborative activities and peer interaction features to address the perceived lack of social engagement reported by some participants. While the study offers valuable insights, several limitations need to be considered. The study has not investigated its long-term influences on learning outcomes. Therefore, future research is recommended to investigate the long-term influence of how well-improved learning outcomes, such as acquired knowledge and skills, can be retained over time. This will provide deeper insights into its effectiveness. While the current study’s findings show that microlearning contributes to increased motivation to learn, further research is recommended to understand how it improves motivation.

## Conclusion

The findings of this study indicate that the MIND model supports effective instructional design and enhances learning outcomes, outperforming the ADDIE model. Learning outcomes were consistent across gender, employment status, and locality within the MIND model group, highlighting the inclusivity and accessibility of instructional design and the microlearning approach. All age groups demonstrated improvement, with younger learners showing particularly significant gains, reflecting their digital fluency. Qualitative reflections complemented the quantitative findings, revealing contributing factors, including media richness, interaction, engagement, self-concept, motivation, and satisfaction. These findings underscore the effectiveness of the MIND model in enhancing learning outcomes and provide practical implications for instructional designers, educators, and policymakers seeking to implement microlearning in lifelong learning and beyond. Overall, the MIND model is an innovative instructional design model that integrates cutting-edge technologies into instructional design, remains adaptable to conventional approaches, and ensures consistency across stages from analysis through assessment & evaluation for continuous improvement.

## Data Availability

Data will be available on a reasonable request from the correspondence author.
